# Reduced ribosomal RNA expression and unchanged ribosomal DNA promoter methylation in oral squamous cell carcinoma

**DOI:** 10.1002/mgg3.783

**Published:** 2019-06-06

**Authors:** Shanshan Ha, Hong Zhou, Mayank Gautam, Yaling Song, Changning Wang

**Affiliations:** ^1^ The State Key Laboratory Breeding Base of Basic Science of Stomatology (Hubei‐MOST) & Key Laboratory of Oral Biomedicine Ministry of Education, School & Hospital of Stomatology Wuhan University Wuhan China; ^2^ State Key Laboratory of Hybrid Rice, College of Life Sciences Wuhan University Wuhan China; ^3^ School of Basic Medicine Tongji Medical College, Huazhong University of Science and Technology Wuhan China

**Keywords:** DNA methylation, epigenetics, oral squamous cell carcinoma, rDNA, rRNA

## Abstract

**Background:**

Ribosomal RNA (rRNA) consists of four non‐coding RNAs, the 28S, 5.8S, 18S, and 5S rRNA. Abnormal expression of rRNA has been found in multiple tumors, and the methylation of rDNA promoter may affect rRNA expression as an epigenetic regulatory mechanism. Oral squamous cell carcinoma (OSCC) is a kind of aggressive tumors which occurs in multiple sites in oral cavity. rRNA expression and the methylation of rDNA promoter in modulating rRNA expression in OSCC maintain unclear. This study aims to investigate the rRNA expression, the methylation status within rDNA promoter, and the underlying mechanism of methylation in regulating rRNA expression in OSCC.

**Methods:**

Twelve primary OSCC and matched normal tissue samples were collected from patients with OSCC. Quantitative real‐time PCR was used to evaluate the rRNA level. HpaII/MspI digestion and bisulfite sequencing were used to investigate the methylation status of rDNA promoter.

**Results:**

Ribosomal RNA levels were suppressed in OSCC as compared with matched normal tissues. HpaII/MspI digestion and bisulfite sequencing showed no significant differences for the methylation of rDNA promoter between the tumor and matched normal tissues.

**Conclusion:**

The methylation in rDNA promoter could not explain for the suppressed rRNA expression in OSCC tissues.

## 1 INTRODUCTION

Oral squamous cell carcinoma (OSCC), a kind of heterogeneous cancer in the squamous epitheliums, is found in 80%–90% of head and neck cancers and is among the 10 most frequent human malignancies (Thompson, 2006; Torre et al., 2015). OSCC behaves as an aggressive tumor which occurs at multiple sites in oral cavity, including tongue, upper and lower gingiva, oral floor, palate, and buccal mucosa. OSCC mostly arises in the context of excessive consumption of alcoholic beverages and tobacco smoking (Kim et al., 2016). OSCC is the major causes of morbidity and mortality in head and neck cancers around the world (Torre et al., 2015). Despite the advancement in cancer research and treatment, the survival rate for OSCC has shown <50% over the last three decades and the overall 5‐year survival rate for patients with OSCC remains the lowest among malignancies (Kim et al., 2016). The poor prognosis of OSCC over the past several years is mostly due to the development of distant metastasis, locoregional recurrences, and new tumors (Hunter, Parkinson, & Harrison, 2005; Leemans, Braakhuis, & Brakenhoff, 2011; Sano & Myers, 2007; Torre et al., 2015). Earlier detection and diagnosis of the OSCC is significant for the earlier treatment.

A hallmark of tumor cells is the increase in size and number of nucleoli (Shiue, Arabi, & Wright, 2010). It was proposed that the size enlargement and the number increasing for nucleoli in cancer cells arose from the hyperactivity of ribosomal RNA (rRNA; Ruggero & Pandolfi, 2003). The rRNA consists of four non‐coding RNAs, the 28S, 5.8S, 18S, and 5S rRNA. Under the action of RNA polymerase I (Pol I), rRNA genes (rDNA) are transcribed to long rRNA precursor (45S pre‐rRNA), which would be further processed into mature 28S, 5.8S, and 18S rRNA (Grummt, 2003). In addition, 5S rRNA is directly transcribed by RNA polymerase III. Previous studies indicated that the expression of 45S pre‐rRNA, 28S, 18S, and 5.8S rRNA were increased in human primary prostate cancers and in C‐MYC‐driven cancers (Drygin, Rice, & Grummt, 2010; Uemura et al., 2012), while rRNA expression was suppressed in CD34+ cells derived from bone marrows of patients with myelodysplastic syndromes (Raval et al., 2012). Different type of cancers exhibiting the rRNA expression variation (activation/suppression) indicates the underlying different mechanism in tumorigenesis. Elucidating the activation or suppression of rRNA and the potential mechanism in tumors would contribute to the earlier diagnosis and intervention.

Recent studies showed that epigenetic mechanisms were involved in Pol I‐directed rRNA gene (rDNA) transcription (Grummt & Pikaard, 2003). Abnormal DNA methylation led to oncogene activation, genetic genomic instability, and tumor suppressor gene silencing, which could result in uncontrolled cell proliferation in tumors (Baylin et al., 2001; Jones & Baylin, 2002; Plass, 2002; Robertson & Wolffe, 2000). DNA methylation modulates rRNA gene transcription in multiple cancer types. Hypomethylation led to the hyperactivation of rRNA gene transcription in human hepatocellular carcinomas (Ghoshal et al., 2004), lung cancer (Lu et al., 2009), and cervical cancer (Zhou et al., 2016), whereas hypermethylation led to the repression of rRNA gene transcription in CD34+ cells derived from patients with myelodysplastic syndromes (Raval et al., 2012).

Despite the increasing body of evidence that modulation (hyperactivity/suppression) in rRNA expression occurs in distinct cancers, there has been limited study on rRNA expression in OSCC cancers and the underlying mechanism remains largely unknown. The role of cytosine methylation in rRNA gene regulation needs to be further explored (Grummt & Pikaard, 2003). In order to clarify the pathogenesis of OSCC and explore the potential target for intervention, the present study aims to investigate the rRNA expression, the methylation status of the CpG islands within the promoter of rDNA, and the possible role of methylation in regulating rRNA expression in OSCC.

## 2 MATERIALS AND METHODS

### 2.1 Ethical compliance and patient samples

The study protocol was approved by the Ethics Committee at School & Hospital of Stomatology, Wuhan University. The surgically resected primary OSCC and matched marginal normal epithelium tissues were obtained from patients at School & Hospital of Stomatology, Wuhan University. The informed consent for publication of their clinical details and/or clinical images was obtained from the patients. Tissues obtained from patients during surgery were immediately frozen and stored at −80°C. All diagnoses were confirmed by pathological examination with hematoxylin and eosin (H&E) staining.

### 2.2 Quantitative real‐time PCR assay

RNA was extracted with TRIZOL (Invitrogen, Carlsbad, CA) obtained from frozen tissues in −80°C. The purified RNA was reverse‐transcribed to cDNA by using a Revert Aid First Strand cDNA Synthesis Kit (Fermentas, Burlington, ON, Canada) following the manufacturer's instructions. Quantitative real‐time PCR (qRT‐PCR) was performed using a StepOne Plus real‐time PCR system (Applied Biosystems, Carlsbad) in the presence of SYBR Green Real‐time PCR Master Mix (TOYOBO, Tokyo, Japan). The thermal cycling conditions were comprised of an initial denature step at 94°C for 2 min, 40 cycles at 95°C for 5 s, 56°C for 15 s, and 72°C for 20 s. Fluorescence data were acquired at the 72°C step and during the melting curve program. *GAPDH* was selected as a reference gene. The relative gene expression was calculated by the 2^(−△△CT)^ method and normalized to *GAPDH*. Quantitative real‐time PCR was repeated three times for each sample from three independent experiments. All primer sequences are shown in Table 1.

**Table 1:** Sequences of primers for quantitative real‐time PCR

Primer	Forward primer (5’−3’)	Reverse primer (5’−3’)
45S rRNA	GAACGGTGGTGTGTCGTT	GCGTCTCGTCTCGTCTCACT
28S rRNA	AGAGGTAAACGGGTGGGGTC	GGGGTCGGGAGGAACGG
18S rRNA	GATGGTAGTCGCCGTGCC	GCCTGCTGCCTTCCTTGG
5.8S rRNA	ACTCGGCTCGTGCGTC	GCGACGCTCAGACAGG
*GAPDH*	CCCCTTCATTGACCTCAACTACAT	CGCTCCTGGAAGATGGTGA
rDNA promoter after HpaII/MspI digestion	TCCGTGTGTGGCTGCGAT	GAGGACAGCGTGTCAGCATAT
rDNA promoter bisulfite sequence	GTTTTTGGGTTGATTAGA	AAAACCCAACCTCTCC

### 2.3 DNA extraction and site‐specific methylation by HpaII/MspI digestion and real‐time PCR examination

Genomic DNA was extracted from the frozen‐matched pairs of OSCC tumor and marginal normal epithelium tissues by a Genomic DNA Mini Preparation Kit with Spin Column (Beyotime, D0061, Shanghai, China), according to the manufacturer's protocol. The DNA samples were separately digested with HpaII and MspI (Thermo, Dalian, China). HpaII and MspI recognize the same restriction site (CCGG) but have different sensitivities to cytosine methylation. HpaII cannot cleave DNA if the cognate restriction sites are methylated, while MspI can cleave every restriction site regardless of its methylation. Then, the methylated and unmethylated rDNA promoters were roughly detected by qRT‐PCR with the digested DNA as a template. After digested by HpaII, there would be more qRT‐PCR products in the samples with high level of methylation than those in the samples with low‐level methylation.

### 2.4 Sodium bisulfite modification and bisulfite genomic sequencing

To determine the methylation level more accurately, we conducted sodium bisulfite modification and bisulfite genomic sequencing. DNA samples were treated with sodium bisulfite in order to convert unmethylated cytosine residues to uracil via deamination using the EZ Methylation‐Gold Kit (Zymo Research, Orange, CA). PCR reactions were performed in a 25‐μl reaction system containing 2 μl bisulfite‐modified DNA, 2 μl 10 × PCR buffer, 2 μl 2.5 mmol/L dNTP mixture, 0.5 μl HiFi Taq‐polymerase, 1 μl of each primer, and 12.5 μl H_2_O. PCR conditions were 95°C for 4 min, 35 cycles of 94°C for 30 s, annealing at 50°C for 45 s, and 72°C for 45 s, followed by a final extension step at 72°C for 10 min. The obtained product was recovered and purified. Purified products were cloned into the pEASY‐T1 cloning vector using the pEASY‐T1 Cloning Kit. Bacteria were plated on LB‐agar containing ampicillin. Plasmid DNA from at least 15 positive clones was sequenced. To ensure that the bisulfite conversion was complete, only clones in which all cytosine residues in non‐CpG dinucleotides had been converted to thymine were included in the analysis.

### 2.5 Statistical analysis

All experiments were independently repeated in triplicates. The quantitative data were presented as the mean ± *SD*; Student's *t* test by SPSS 21 software was used for the comparison between the two groups. The relative levels of rRNA expression in OSCC and the paired normal control epithelial tissues were analyzed using the paired *t* test. Quantitative real‐time PCR analysis on the rDNA promoter using HpaII/MspI restriction enzymes was measured by the independent *t* test. Quantitative analysis on methylation density at each CpG and all CpGs in individual OSCC and matched normal oral epithelial tissues was calculated by determining methylation percentage for each CpG marker in the clones from each subject. Then, for each CpG marker, the difference between 12 methylation percentages in OSCC and 12 methylation percentages in normal tissues was analyzed using the independent *t* test. The differences were considered significant when *p* < 0.05.

## 3 RESULTS

### 3.1 Patient data

**Figure 1:**
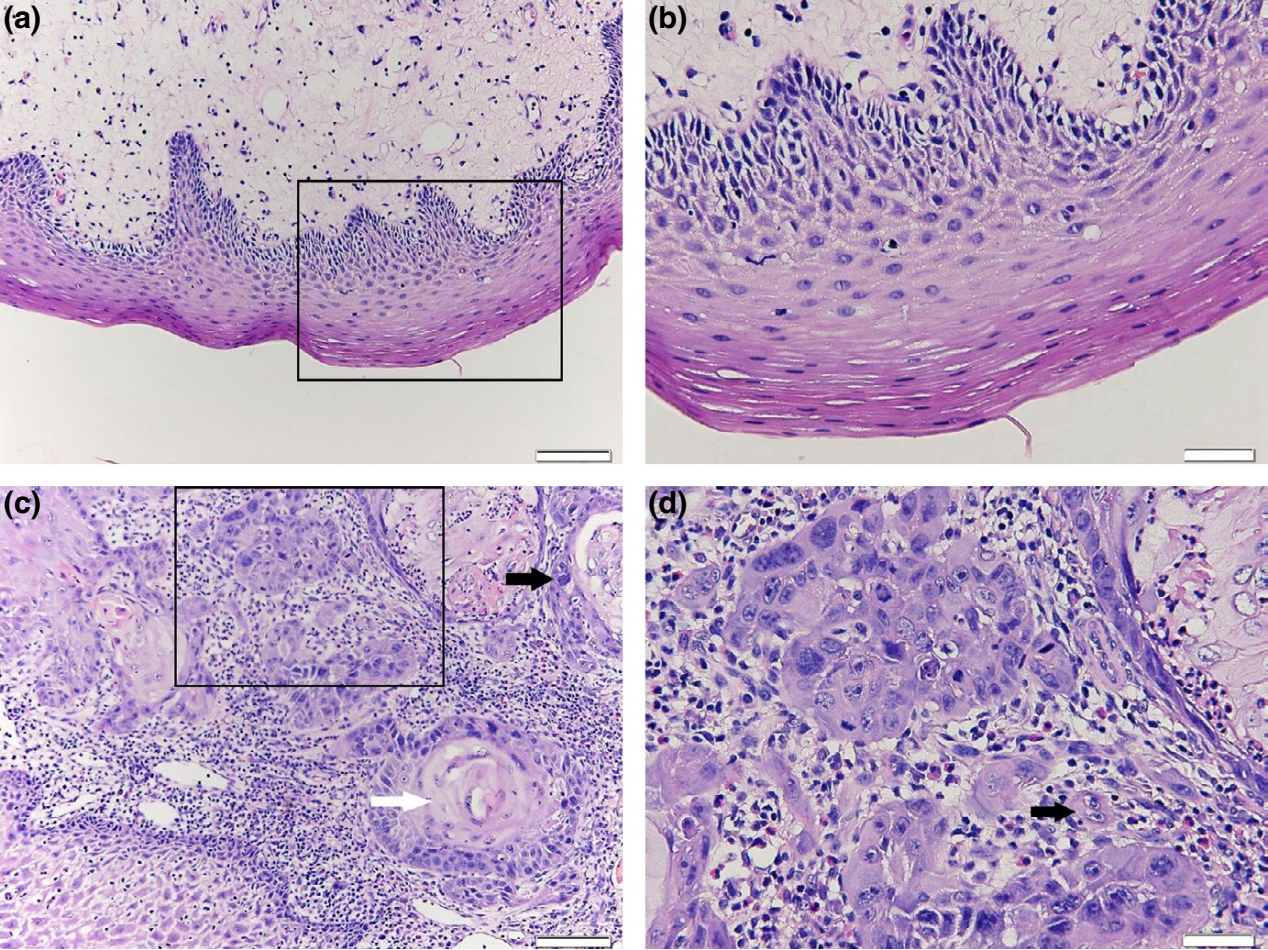
Hematoxylin and eosin (H&E) staining showed abnormalities in oral squamous cell carcinoma (OSCC). (a) Normal oral epithelial tissue, bar = 50 μm; (b) A magnification of the square in (a). bar = 20 μm; (c) HE staining in OSCC tissues shows squamous cell lumps with abnormal proliferation; the white arrow points to a keratin pearl in the center of the cancer nest and the black arrow indicates the heterotypical change in an cell, bar = 50 μm; (d) A magnification of the square in (c), the size, shape, and dyeing of the nucleus are quite different to normal tissues; the arrow indicates a binucleated cell, bar = 20 μm

The primary OSCC and matched marginal normal tissue samples were obtained from 12 patients with OSCC. Clinical and pathological features for each patient are showed in Table 2. Ever smoker was defined as smoking more than 100 cigarettes in his lifetime. Patients were considered as ever drinkers if they drink alcohol at least once a week for one year or more during his lifetime (Dahlstrom et al., 2008). Six out of 10 males were ever drinkers and eight out of them were ever cigarette smokers, six of whom used alcohol and cigarette both. The other two female patients did not smoke or drink alcohol. The pathological grades for each sample were determined according to the WHO classification: Grade 1, well‐differentiated squamous cell carcinoma (SCC) is similar to normal squamous epithelium, including basal cells varied in number and squamous cells with intercellular bridges, obvious keratinization, few mitotic phases, rare atypical mitosis and multinucleated cells, inapparent nuclear, and cellular pleomorphism; Grade 2, moderate‐differentiated SCC has distinctive nuclear pleomorphism and division, including abnormal nuclear division, unusual keratinization, and inapparent intercellular bridges; Grade 3, poorly differentiated SCC is dominated by immature cells with plenty of normal or abnormal mitosis, little keratinization, and very few intercellular bridges (Thompson, 2006). By pathological examination with H&E staining, the atypical nuclear phenomenon with double nucleus or multinucleus and the enlarged cells were presented in OSCC tissues, compared with the normal oral epithelial cells (Figure 1).

**Table 2:** Clinical and pathological characteristics of the patients

Patient no.	Age (years)	Sex	Occurrence site	Cigarette	Alcohol	Margin	Pathological grade
1	48	Male	Tongue	Yes	Yes	Negative	2
2	63	Female	Buccal mucosa	No	No	Negative	1–2
3	55	Male	Root of tongue	No	No	Negative	2–3
4	52	Male	Buccal mucosa	Yes	Yes	Negative	1–2
5	50	Male	Buccal mucosa	Yes	Yes	Negative	2
6	59	Male	Root of tongue	No	No	Negative	2–3
7	37	Male	Oral floor mucosa	Yes	No	Negative	1–2
8	72	Female	Buccal mucosa	No	No	Negative	1
9	41	Male	Tongue	Yes	No	Negative	1
10	57	Male	Tongue	Yes	Yes	Negative	2
11	48	Male	Tongue	Yes	Yes	Negative	1
12	55	Male	Tongue	Yes	Yes	Negative	1

Note

Margin negative means the edge of the excised tissues are non‐neoplastic regions.

### 3.2 Expression of rRNA transcripts in OSCCs

**Figure 2:**
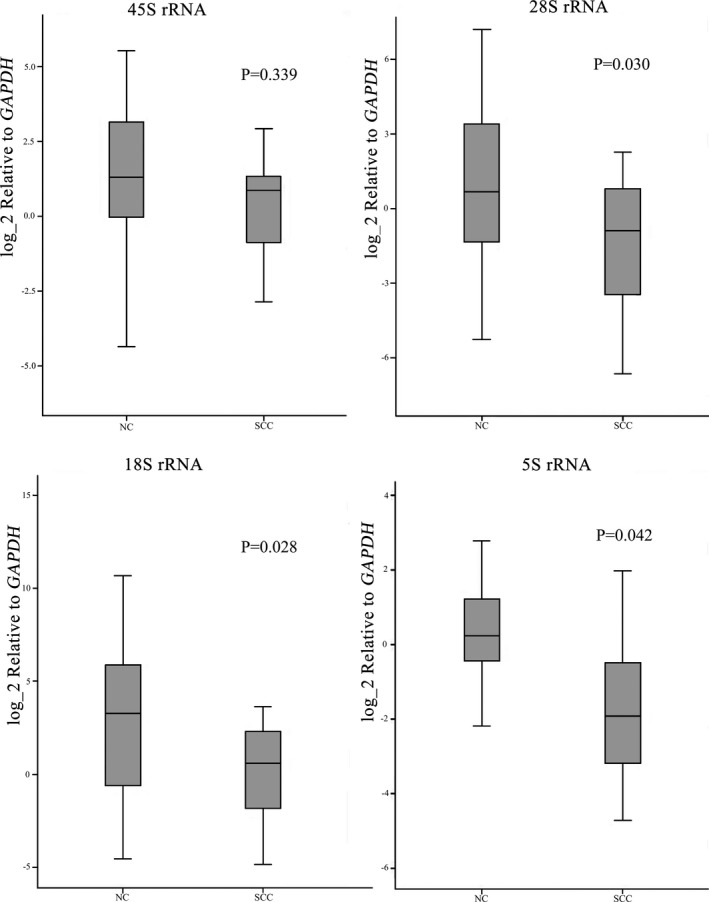
Box plots showed the relative levels of ribosomal RNA expression in oral squamous cell carcinoma (OSCC) (SCC) and the paired normal control tissues (NC)

Total RNA was isolated from the frozen tumor and matching normal tissue samples obtained from 12 OSCC patients. Quantitative real‐time PCR analysis was performed for the 45S precursor, and the 28S,18S and 5.8S mature rRNA forms. The results showed that RNA expression for 28S (*p* = 0.030), 18S (*p* = 0.042), and 5.8S (*p* = 0.028) mature rRNA was significantly lower in OSCC tissues than those in normal control tissues, and a decreasing trend without statistical difference for 45S precursor rRNA was presented in OSCC tissues (Figure 2). The results indicated that the rRNA transcription was suppressed in OSCC compared with the paired normal control epithelial tissues.

### 3.3 Methylation of the rDNA promoter in OSCC

**Figure 3:**
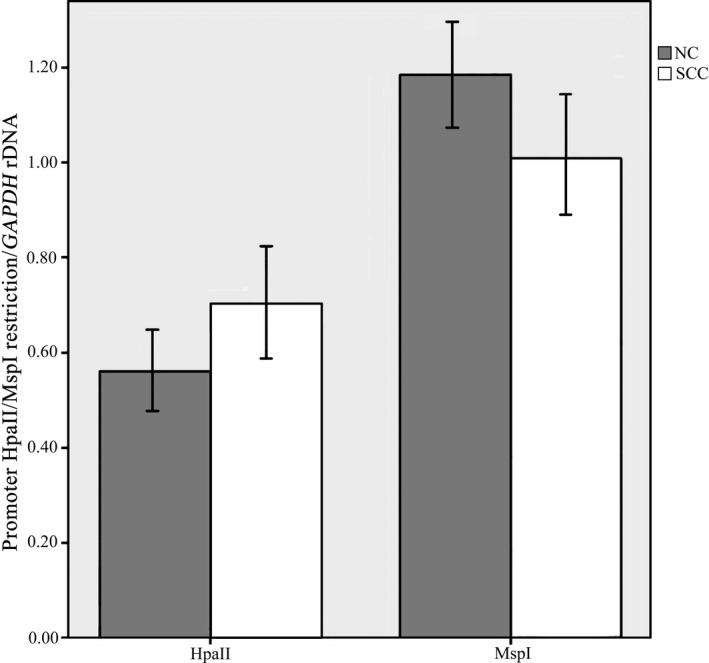
Quantitative real‐time PCR analysis of the rDNA promoter using HpaII/MspI restriction enzymes in oral squamous cell carcinoma (OSCC) (SCC) and normal control tissues (NC) showed the amounts of amplified fragments in the rDNA promoter had no significant difference between OSCC and the normal control epithelial tissues

The rRNA gene (rDNA) promoter is highly enriched in CpG dinucleotides. In order to explore whether the methylation of the rDNA promoter could influence the rRNA expression level in OSCC, the level of methylation was examined by HpaII/MspI digestion and bisulfite genome sequencing. The qRT‐PCR results after HpaII/MspI digestion showed that the amounts of amplified fragments in the rDNA promoter with four CCGG restriction sites had no significant difference between OSCC and the normal control tissues (Figure3), which indicated the similar methylation level among OSCC and normal tissues.

**Figure 4:**
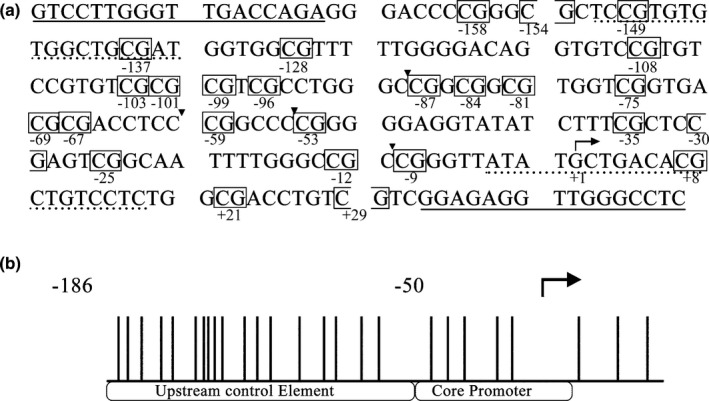
(a) The published sequence of the human rDNA promoter (GenBank accession number U13369.1); the primer sequences for bisulfite sequencing are indicated as underlines; the PCR primer sequences after enzyme digestion are indicated as dotted underlines; the target sites of HpaII/MspI restriction enzymes are marked by a triangle icon between nucleotides and CpG dinucleotide are boxed with the location marked relative to the +1 transcription start site (arrow). (b) A schematic of CpG dinucleotide in the rDNA promoter; vertical lines indicate sites of the CpG dinucleotide; the arrow shows the +1 transcription start site

**Figure 5:**
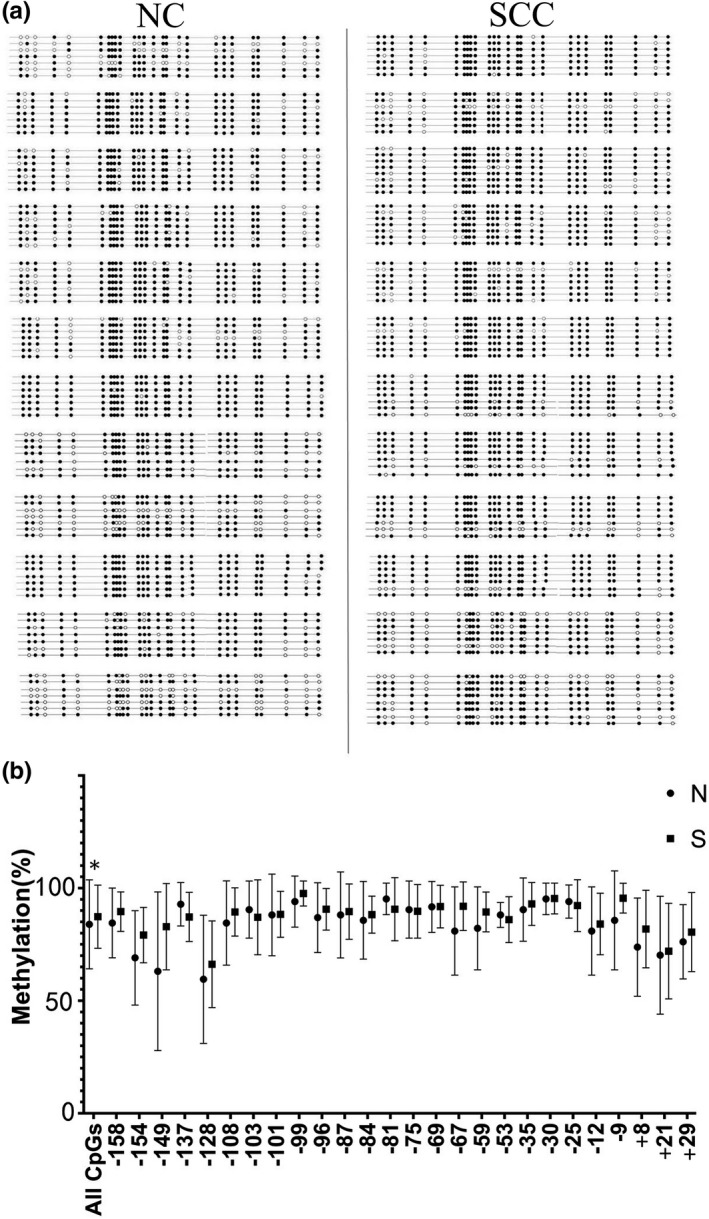
The methylation status of each CpG base pair in ribosomal RNA (rRNA) gene promoter in OSCC and the matched normal oral epithelial tissues from 12 patients with OSCC. (a) Part of the sodium bisulfite mapping of the rRNA gene promoter in OSCC tissues and matched normal oral epithelial tissues. The filled circle denotes methylated CpG dinucleotide and the open circle represents unmethylated CpG dinucleotide. (b) Quantitative analysis of the methylation density with respect to the +1 site of the rRNA promoter in individual tumors and normal oral epithelial tissues. N, normal tissue; S, OSCC tissue

Further evaluation on the methylation status of the CpG island within the rRNA gene promoter was analyzed by bisulfite genome sequencing. The changes of the sequence were analyzed by comparing with the published reference sequence of the human rDNA promoter region (GenBank accession number: U13369.1). Twenty‐six CpG dinucleotides were contained in a 229‐bp rRNA gene promoter region including the upstream control element and the core promoter sequence relative to the +1 transcription start site (Figure 4a,b). Sodium bisulfite mapping the methylated sites in 26 CpG dinucleotides of the rRNA gene promoter in OSCC tissues and normal oral epithelial tissues was showed in Figure 5a. The percentage of methylated clones and unmethylated clones at each position were showed in Figure 5b. There were no significant differences for the methylation at each CpG between OSCC and matched normal oral epithelial tissues, but the methylation of all CpGs made a statistical difference (*p* = 0.014). The results indicated that the methylation of the rRNA gene promoter in OSCC did not explain the lower expression of rRNA in tumor tissues.

## 4 DISCUSSION

Human genome contains 300–400 copies of rRNA genes arranged in predominantly tandem repeated arrays at the secondary constrictions of acrocentric chromosomes 13, 14, 15, 21, and 22, but not all of the genes are actively transcribed (Schmickel, 1973). These genes are responsible for the production of 60% of cellular RNA, which are essential for ribosome biogenesis (Warner, 1999). The altered rRNA synthesis occurred very commonly in cancer cells (Drygin et al., 2010). rRNA genes hyperactivity or suppression are associated with the development of different kinds of tumors (Nguyen, Raval, Garcia, & Mitchell, 2015).

The expression level of rRNA varied in different types of cancers. In colorectal cancer and lung cancer, in order to meet the demand for more ribosomal production and protein synthesis in tumors, compared with pair‐matched normal tissues, the expression of rRNA was increased in tumor tissues (Lu et al., 2009; Tsoi et al., 2017). In breast cancer tissues, using the geometric mean of rRNA expression (GM‐rRNA) as a reference value, 18S rRNA/GM‐rRNA ratio was significantly higher, while the 5.8S rRNA/GM‐rRNA ratio was significantly lower in tumor samples than in matched normal tissues (Karahan et al., 2015). The decreased expression of rRNA in CD34+ cells from patients with myelodysplastic syndromes may reduce the synthesis of ribosomal proteins leading to defective hematopoiesis and bone marrow failure (Raval et al., 2012). A recent study found that the copy number of 45S rDNA in head and neck squamous cell carcinoma tissues is reduced compared with adjacent normal tissues (Wang & Lemos, 2017). However, there is still no report on rRNA expression in OSCC tissues. Consistent with the findings in myelodysplastic syndromes, the present study showed the significant suppression in expression of 5.8S, 18S, and 28S mature rRNA in OSCC tissues.

The underlining regulating mechanism for the transcription of rRNA genes are associated with the number of active rRNA genes, which can be adjusted by epigenetic mechanisms (Jacob & Ghosh, 1999). It was proposed that DNA hypermethylation and histone deacetylation contributed to rDNA silencing (Chen & Pikaard, 1997). Previous studies showed that in human cervical cancer tissues and hepatocellular carcinoma tissues, DNA methylation level was decreased in rRNA gene promoter region and the RNA expression level was increased in human cervical cancer and hepatocellular carcinomas (Ghoshal et al., 2004; Zhou et al., 2016). Inconsistent with these previous findings, the present study showed the DNA methylation status of rDNA promoter region was essentially unchanged in OSCC tumor samples as compared with matched normal samples. It was also reported that DNA methylation level remained unchanged but the expression of 45S rRNA was significantly increased in prostate cancers (Uemura et al., 2012). One possible explanation for the present finding is that other underlying mechanism apart from DNA methylation might be involved in the decrease of rRNA transcriptional level in OSCC.

On the other hand, environmental factors exert the indispensable influence during carcinogenesis. Among a variety of exogenous factors, tobacco and alcohol regular intake are closely associated with many kinds of cancers. The possible mechanism was that these predisposing factors might lead to a wide range of genetic and epigenetic events that promote tumor development and progression (Bosse et al., 2012; Seitz & Stickel, 2007). In the present study, eight out of 12 patients were ever cigarette smokers and alcohol drinkers, six of whom consume both cigarette and alcohol. It was showed that smoking altered DNA methylation patterns and gene expression in lung tissues of non‐small cell lung neoplasms (Freeman, Chu, Hsu, & Huang, 2016). Methylation disorders were found in cancers associated with alcohol consumption (Seitz & Stickel, 2007). In the present study, in patients with tobacco and alcohol regular intake, histologically normal cells may have epigenetic alterations in the tissues surrounding the tumor, and these cells might have a potential malignant transformation but have not yet developed a fully neoplastic phenotype. Thereby, it could be speculated that no significant difference in the methylation between OSCC tissues and matched normal tissues might due to the methylation change in both tissues at the same time because of the use of alcohol and tobacco.

Overall, within the limitation of this study, the results suggested that the rRNA levels are significantly suppressed in human OSCC, and that the methylation in the rRNA gene promoter might not explain the decreased rRNA expression in OSCC tissues. Further studies are needed to explore other underlying mechanism involving in suppressing rRNA transcription levels in human OSCC.

## CONFLICT OF INTEREST

None.
